# Offspring subcutaneous adipose markers are sensitive to the timing of maternal gestational weight gain

**DOI:** 10.1186/s12958-015-0009-0

**Published:** 2015-03-08

**Authors:** Linda Giblin, Christian Darimont, Patricia Leone, Louise B McNamara, Florence Blancher, Donagh Berry, Eurídice Castañeda-Gutiérrez, Peadar G Lawlor

**Affiliations:** Teagasc Food Research Centre, Moorepark, Fermoy, Co.Cork, Ireland; Nestlé Research Centre, Nutrition & Health Research Department, Vers-Chez-les-Blanc, Lausanne, Switzerland; Animal and Grassland Research and Innovation Centre, Teagasc, Moorepark, Fermoy, Co. Cork, Ireland

**Keywords:** Maternal food intake, Gestational weight gain, Subcutaneous adipose tissue

## Abstract

**Background:**

Excessive maternal weight gain during pregnancy impacts on offspring health. This study focused on the timing of maternal gestational weight gain, using a porcine model with mothers of normal pre-pregnancy weight.

**Methods:**

Trial design ensured the trajectory of maternal gestational weight gain differed across treatments in early, mid and late gestation. Diet composition did not differ. On day 25 gestation, sows were assigned to one of five treatments: Control sows received a standard gestation diet of 2.3 kg/day (30 MJ DE/day) from early to late gestation (day 25–110 gestation). E sows received 4.6 kg food/day in early gestation (day 25–50 gestation). M sows doubled their food intake in mid gestation (day 50–80 gestation). EM sows doubled their food intake during both early and mid gestation (day 25–80 gestation). L sows consumed 3.5 kg food/day in late gestation (day 80–110 gestation). Offspring body weight and food intake levels were measured from birth to adolescence. Markers of lipid metabolism, hypertrophy and inflammation were investigated in subcutaneous adipose tissue of adolescent offspring.

**Results:**

The trajectory of gestational weight gain differed across treatments. However total gestational weight gain did not differ except for EM sows who were the heaviest and fattest mothers at parturition. Offspring birth weight did not differ across treatments. Subcutaneous adipose tissue from EM offspring differed significantly from controls, with elevated mRNA levels of lipogenic (*CD36*, *ACACB* and *LPL*), nutrient transporters (*FABP4* and *GLUT4*), lipolysis (*HSL* and *ATGL*), adipocyte size (*MEST)* and inflammation (*PAI-1)* indicators. The subcutaneous adipose depot from L offspring exhibited elevated levels of *CD36, ACACB, LPL, GLUT4* and *FABP4* mRNA transcripts compared to control offspring.

**Conclusions:**

Increasing gestational weight gain in early gestation had the greatest impact on offspring postnatal growth rate. Increasing maternal food allowance in late gestation appeared to shift the offspring adipocyte focus towards accumulation of fat. Mothers who gained the most weight during gestation (EM mothers) gave birth to offspring whose subcutaneous adipose tissue, at adolescence, appeared hyperactive compared to controls. This study concluded that mothers, who gained more than the recommended weight gain in mid and late gestation, put their offspring adipose tissue at risk of dysfunction.

## Background

Thirty per-cent of 7 year old children in Europe are overweight or obese [1]. Childhood obesity tracks to adulthood [2]. An obese child has more than a 60% chance of being obese in adulthood [2]. Obese girls grow up to be obese women who give birth to babies who are destined to be obese themselves [2]. Although the root cause of obesity is excess caloric intake compared with expenditure, it is now accepted that an adverse (excess or restricted) nutrient supply in early life is linked to obesity and obesity-related disorders in later life [2-5]. Prenatal overnutrition can permanently change offspring energy homeostasis [5], appetite drive and adipocyte function [6,7]. Adipocytes are fat storage cells playing a major role in metabolic haemostasis. In obese individuals, adipocytes are dysfunctional [8].

The effects of maternal overnutrition on offspring are particularly relevant in modern day life, where high maternal Body Mass Index (BMI) and/or a large maternal gestational weight gain are common place [2,3,9]. Rats fed a high fat palatable diet gave birth to offspring with greater adiposity, increased serum glucose and elevated lipogenic activity in white adipose tissue at adulthood [10]. In sheep experimental models, maternal overnutrition prior to and during pregnancy gave birth to offspring of similar birth weight. However by adulthood, these offspring had increased fat mass % (20.8% versus 16.5% for controls), increased feed intake by 10% and increased glucose-insulin baselines when fed *ad libitum* [11].

The timing of maternal malnutrition is also important for offspring health status [12]. Women with a healthy weight pre-pregnancy, BMI of 18.5-24.9, are advised to gain 11.5-16 kg during pregnancy which includes a maximum of 2 kg in the 1st trimester followed by 0.36-0.45 kg per week thereafter. Epidemiological data from episodes of famine have demonstrated that maternal undernutrition during the embryonic and placental stage affects offspring cardiovascular health [12,13]. Maternal undernutrition during mid gestation results in offspring renal and adipose tissue dysfunction [12,13]. Maternal dietary restrictions imposed in late gestation compromise offspring metabolism and subcutaneous to visceral adipose ratios [13,14].

Adipose tissue is particularly susceptible to developmental programming as a result of maternal nutrition *in utero* [6,7]*.* Maternal malnutrition is capable of altering adipocyte number, size, maturation and capacity to store fat [15]. Any permanent alteration to adipocyte number or function *in utero* has serious consequences for adipose tissue for life. Adipose tissue is an unusual tissue as it is capable of unlimited growth in adulthood [16]. The subcutaneous adipose depot is the primary and initial fat storage facility in the healthy weight individual. When it’s capacity to store fat is exceeded, fat storage spills over into visceral adipose depots [17]. Visceral and subcutaneous adipocytes in obese individuals exhibit dysregulation of lipogenesis and lipolysis, altered endocrine function and increased level of macrophage infiltration [8,18].

A dysfunctional adipocyte due to maternal malnutrition does not necessarily manifest as an alteration in offspring body weight [14,16], rather body composition (% fat mass) may be altered [14,19]. Indeed an underlying adipocyte dysfunction may only become apparent later in life and/or when the offspring is challenged postnatally with an abundant supply of highly palatable, high fat food [10,19].

As rodents self limit their food intake, rodent models for maternal overnutrition must rely on either modifications to the diet composition or gastric cannulation [20]. The pig is an alternative animal model. Pigs do not generally exhibit self limitation of food intake and will become overweight when given *ad libitum* access to food [21]. In addition, pigs display a meal eating pattern, are omnivores and have similar metabolism, digestive tract, cardiovascular system and proportional organ sizes to humans [22]. Pigs, however, are born with only 2% body fat compared to 15% in humans [23]. Piglets will rapidly reach 15% body fat within 28 days of birth [24].

The aim of this study was to investigate whether extra maternal nutrition at different windows of gestation can alter markers of lipid metabolism, hypertrophy and inflammation in subcutaneous adipose tissue of offspring. Subcutaneous adipose tissue is the largest fat depot in young pigs [22]. The tissue was harvested at adolescence as juvenile adipose tissue may be a poor predictor of later function [25].

Selecting sows of normal and similar pre-pregnancy weight allowed us to focus solely on gestational weight gain. The trial design attempted to keep overall maternal backfat increases across treatments close to normal ranges, whilst differing the amount of weight gain in early, mid and late pregnancy. Previous results from this trial indicated that offspring from mothers who were overfed in early gestation had modified muscle composition with reduced intramuscular fat levels and increased type IIA muscle fibres in their semitendinosus muscle [26,27]. The porcine foetal subcutaneous adipose tissue undergoes rapid development in mid to late gestation (day 50 to parturition (day 115)) with lipid deposition commencing by day 60 gestation, opening of adipocyte cluster by day 75 and growth of configured adipocytes from day 105 gestation [28]. Based on this timeline, our hypothesis was that increased food intake by the mother in mid to late gestation would have the greatest effect on offspring adipocyte function.

## Methods

### Animals and treatments

This experiment complied with the EU Council Directive 91/630/EEC, which lays down minimum standards for the protection of pigs [29]. It also complied with the EU Council Directive 98/58/EC, concerning the protection of animals kept for farming purposes [29]. The trial was conducted in accordance with the International Guiding Principles for Biomedical Research Involving Animals as issued by the Council for the International Organizations of Medical Sciences in 1985. The trial commenced and finished in 2006.

A total of 65 multiparous Landrace x Large White crossbred sows were artificially inseminated using the pooled semen from 7 Hylean Large White boars (Hermitage AI, Co. Kilkenny, Ireland). The ingredient composition and chemical analysis of the diets are listed in Table 1. As per recommendations [30], all sows were fed 1.8 kg/day (23.5 MJ DE/day) of a standard gestation diet from service to day 25 gestation.

**Table 1:** **Ingredient composition and nutrient content of experimental diets on a meal equivalent basis (g/kg)**

**Diet type**	**Gestation**	**Lactation**	**Starter** ^**a**^	**Link** ^**a**^	**Weaner**	**Finisher**
Wheat	0	424	ndc	ndc	455	404
Barley	893	350	ndc	ndc	225	364
Soya 50	75	160	ndc	ndc	180	200
Full fat soya	0		ndc	ndc	100	0
Soya oil	10	40	ndc	ndc	10	10
Mineral and vitamins^b^	1.5	1.5	ndc	ndc	3	1
Lysine HCl^c^	0.5	2	ndc	ndc	4	3
DL-Methionine^c^	0	0.7	ndc	ndc	2	0.8
L-Threonine^c^	0	0.8	ndc	ndc	1.5	1
Di-calcium phosphate	5	5	ndc	ndc	5	
Limestone flour	11	12	ndc	ndc	11	13
Salt	4	4	ndc	ndc	3	3
Pulmotil^d^	0	0	ndc	+	0	0
Phytase 5000,^e^ IU/g	0.1	0.1	ndc	ndc	0.1	0.1
**Chemical composition, g/kg**						
Dry matter	871	873	870	870	872	870
Crude Protein	132	158	200	200	196	178
Fat	31	56	90	75	43	27
Crude fibre	45	35	25	30	36	37
Ash	44	46	60	60	50	44
Lysine^f^	6.2	9.1	16	15	13.1	11.1
Digestible energy,^f^ MJ/kg	13	14.2	16.3	15.4	14.1	13.7

Sow, weaner and finisher diets were manufactured onsite.

Starter and link diets were manufactured by Devenish Nutrition (Belfast, Northern Ireland).

^a^Commercial diets for which the ingredient composition was not disclosed (ndc).

^b^Provided per kilogram of complete diet.

Gestation and lactation diets: Cu, 38 mg; Fe, 70 mg; Mn, 62 mg; Zn, 80 mg; I, 0.6 mg; Se, 0.2 mg; vitamin A, 10000 IU; vitamin D_3_, 1000 IU; vitamin E, 100 IU; vitamin K, 2 mg; vitamin B_12_, 15 μg; riboflavin, 5 mg; nicotinic acid, 12 mg; pantothenic acid, 10 mg; choline chloride, 500 mg; Biotin, 200 μg; Folic acid, 5 mg; vitamin B_1_, 2 mg and vitamin B_6_, 3 mg.

Weaner diet: Cu, 175 mg; Fe, 140 mg; Mn, 47 mg; Zn, 120 mg; I, 0.6 mg; Se, 0.3 mg; vitamin A, 6000 IU; vitamin D_3_, 1000 IU; vitamin E, 100 IU; vitamin K, 4 mg; vitamin B_12_, 15 μg; riboflavin, 2 mg; nicotinic acid, 12 mg; pantothenic acid, 10 mg; choline chloride, 250 mg; vitamin B_1_, 2 mg; vitamin B_6_, 3 mg; and endox, 60 mg.

Finisher diet: Cu, 100 mg; Fe, 40 mg; Mn, 31 mg; Zn, 80 mg; I, 0.3 mg; Se, 0.2 mg; vitamin A, 2000 IU; vitamin D_3_, 500 IU; vitamin E, 40 IU; vitamin K, 4 mg; vitamin B_12_, 15 μg; riboflavin, 2 mg; nicotinic acid, 12 mg; pantothenic acid, 10 mg; vitamin B_1_, 2 mg and vitamin B_6_, 3 mg.

^c^Synthetic amino acids.

^d^Link diet contained 200 mg Tilmicosin per kg of feed provided from Pulmotil G100 (Eli Lilly, Basingstoke, Hampshire, England).

^e^Sow, weaner and finisher diets contained 500 FTU phytase per kg finished feed from Natuphos 5000 (BASF, Ludwigshafen, Germany).

^f^Calculated values.

On day 25, sows were assigned to one of five treatments balanced for parity and weight (Table 2). Diet composition did not differ across treatments. Control (C) sows followed the feed intake recommendations for the gestating sow which included a standard gestation diet of 2.3 kg/day dry matter basis (30 MJ DE/day) from day 25 to day 110 gestation (Table 2) [30,31]. E sows received an increased feed allowance to 4.6 kg/day (60 MJ DE/day) early in gestation, from day 25 to day 50 gestation. M sows received 4.6 kg food/day in mid-gestation, from day 50 to day 80 gestation. EM sows received 4.6 kg/day in early and mid gestation, from days 25 to 80 gestation. L sows received 3.5 kg food/day (45.5 MJ DE/day) in late gestation, from day 80 to 110 gestation. At the end of an increased feeding period, sow feed allowance returned to 2.3 kg/day (Table 2). At day 110, approximately 5 days before parturition, all sows were transferred onto a lactation diet of 1.8 kg/day (25.6 MJ DE/day) in preparation for parturition and lactation (Table 2). Sows consumed all food allocations received. *Ad libitum* for pregnant sows approximates 4.6 kg food intake per day [26] with *ad libitum* feeding in late gestation reducing to 3.5 kg/day. The number of sows included in the analysis was 10 C sows, 15 E sows, 13 M sows, 12 EM sows and 11 L sows.

**Table 2:** **Food intake of sows during gestation**

		**Sow treatment** ^**a**^ **: Food intake per day** ^**b**^				
**Gestation days**	**% gestation completed**	**C**	**E**	**M**	**EM**	**L**
Days 1-24	0-20.8%	1.8 kg (23.5 MJ DE/day)	1.8 kg (23.5 MJ DE/day)	1.8 kg (23.5 MJ DE/day)	1.8 kg (23.5 MJ DE/day)	1.8 kg (23.5 MJ DE/day)
Days 25-50	21.7-43.5%	2.3 kg (30 MJ DE/day)	4.6 kg (60 MJ DE/day)	2.3 kg (30 MJ DE/day)	4.6 kg (60 MJ DE/day)	2.3 kg (30 MJ DE/day)
Days 50-80	43.5-69.6%	2.3 kg (30 MJ DE/day)	2.3 kg (30 MJ DE/day)	4.6 kg (60 MJ DE/day)	4.6 kg (60 MJ DE/day)	2.3 kg (30 MJ DE/day)
Days 80-110	69.6-95.7%	2.3 kg (30 MJ DE/day)	2.3 kg (30 MJ DE/day)	2.3 kg (30 MJ DE/day)	2.3 kg (30 MJ DE/day)	3.5 kg (46 MJ DE/day)
Days 110-115	95.7-100%	1.8 kg (25.6 MJ DE/day)	1.8 kg (25.6 MJ DE/day)	1.8 kg (25.6 MJ DE/day)	1.8 kg (25.6 MJ DE/day)	1.8 kg (25.6 MJ DE/day)

^a^C were Control Sows that received 2.3 kg/day food from day 25 to day 110 gestation, E sows consumed 4.6 kg/day food in early (days 25 to 50) gestation, M sows received 4.6 kg/day in mid gestation (days 50 to 80), EM sows received 4.6 kg/day in early and mid gestation (days 25 to 80) and L sows received 3.5 kg/day from days 80 to 110 of gestation.

^b^Food intake per day is expressed as kg of food and as digestible energy (DE).

At parturition, the progeny from each sow were weighed individually and tagged for identification purposes. Within each treatment, litter size was standardised to 10 piglets per litter by cross fostering. All litters received a starter diet (Creep fed) from day 12 post-natal to weaning (day 28 post-natal). At weaning, three same gender pigs from each litter were selected based on birth weight category (light, medium and heavy). The absolute lightest, heaviest and medium weight pigs established within each litter were selected. Therefore, at weaning, a total of 240 pigs (120 females and 120 entire males) were weighed and penned individually. By day 77 this number was reduced to 180 pigs, due to housing restrictions. This was achieved by removing 12 pigs in each treatment (all pigs from 2 randomly selected litters within a treatment). Pigs were fed 3 times daily in the first week and *ad libitum* thereafter. Food intake was recorded weekly. Pigs were weighed at birth, at weaning (day 28) and at days 41, 55, 76, 118 and 159. Housing conditions for sows and pigs are described previously [32].

### Carcass measurements

Pigs were transported 107 km to the abattoir and killed by bleeding after CO_2_ stunning. Muscle depth and backfat thickness, at 6 cm from the edge of the split back at the level of the 3rd and 4th last rib, were measured using a Hennessy grading probe (Hennessy and Chong, Auckland, New Zealand). Cold carcass weight was estimated as the weight of the hot eviscerated carcass, (minus tongue, bristles, genital organs, kidneys, flare fat and diaphragm) 45 min after harvest x 0.98. Formula for estimate lean meat content (%) = 60.30 - 0.847x + 0.147y, where x = fat depth (mm); y = muscle depth (mm) [33].

### Tissue sampling, RNA extraction and cDNA synthesis

Eighteen representative pigs from each treatment (9 female (3 light, 3 medium, 3 heavy birthweight) and 9 males (3 light, 3 medium, 3 heavy birthweight)) were selected randomly at slaughter for adipose tissue sampling*.* Subcutaneous adipose tissue (0.5 cm in depth) was excised from at the backfat measurement position, immersed in RNA-later (Ambion Inc., U.S.A.) and stored at 4°C overnight. Within 24 hours of harvest, these subcutaneous adipose samples were transferred to −80°C for archival storage. Total RNA was extracted by homogenisation of approximately 75 mg adipose tissue using the Agencourt RNAdvance Tissue protocol according to the manufacturer’s instructions (Beckmann Coutler, U.S.A.) which included a *DNAase1* treatment (20units/sample) step. Quality and quantity of total RNA was determined by optical density readings at 260 nm and 280 nm using a Nanodrop ND-1000 (Thermo scientific, USA) and by the Agilent Bioanalyzer system (Agilent, U.S.A). OD260:280 ratios were all above 1.8. RNA extractions were performed in duplicate for each adipose tissue sample. Total RNA samples were stored at −20°C. Total RNA (0.3 μg) was reverse transcribed into cDNA using the PrimeScript 1st strand cDNA synthesis kit (Takara, Japan) in the presence of 20units *RNAase* Inhibitor, according to manufacturer’s instructions into a final volume of 20 μl and stored at −20°C. Reverse transcription reactions were performed in duplicate on each RNA sample.

### Absolute quantification by real-time PCR

Table 3 lists the Genbank accession numbers, primer sequences, annealing temperatures and amplicon sizes of the porcine target RNA. Real time quantitative PCR analyses was performed following MIQE standards [34]. Complementary DNA (1 μl of 1/2.5 dilution of cDNA synthesis reaction), sense and antisense primers (0.45 μM each) was added to SYBR Green 1 PCR core reagents (Applied Biosystems, Life Technologies, U.S.A.) to a final volume of 10 μl using in an ABI prism 7900HT Sequence Detection system Instrument (Applied Biosystems, Life Technologies, U.S.A.). The thermal cycling protocol was as follows: 2 min at 50°C, 1 cycle of denaturation at 95°C for 10mins followed by 40 cycles of amplification 95°C for 15 sec and 60°C for 1 min. Cycle threshold (C_T_) values were the means of at least duplicate experiments. Data were discarded and the experiment repeated if there was 1 cycle difference in Ct value within duplicates on the same plate. A ‘no DNA’ template was run as a negative control on each plate. A melting curve analysis was performed to ascertain single product specific melting temperatures. Complementary DNA samples were pooled to generate an inter plate control for each gene. The real time efficiency of each gene was calculated using a 7 point 4 fold dilution series of cDNA. The efficiencies ranged from 90-100%. A gene that encodes a component of the 60S subunit ribosomal protein, *rPLPO*, was stably expressed in our samples and therefore selected as the reference target. Inter assay and intra-assay variation was less than 5%. For each sample, the relative amount of target was calculated by the 2^–ΔCT^ formula (using the comparative C_T_ method [35]) where ΔC_T_ = C_T_ Target - C_T_ reference. Primers for real-time PCR were designed across intron/exon boundaries where possible, to prevent amplification from porcine genomic DNA.

**Table 3:** **Gene names, primers, genbank accession numbers, annealing temperature and size of amplicons**

**Gene**	**Primer sequence (5′-3’)**	**Genbank**	Ta*	**Amplicon size (bp)**
		**Accession No.**	**(°C)**	
*MEST (Peg-1)*	TGGAGGCGTGCTGTCACCCA	NM_001128471	60	72
	ACTGGGGTGAGACCCCGAGAG		59	
*LEP (Leptin)*	TCCAGGATGACACCAAAACCCTCA	NM_213840.1	58	95
	GGTGACCCTCTGTTTGGAGGAGACA		60	
*PPARGC1 (PGC-1α)*	GGCAATTGAAGAACGTCGTGT	NM_213963.1	60	74
	GGTCCCTCAGTTCTGTCCGT		61	
*CASP1 (Caspase-1; ICE; IL1BC; P45)*	GGAGACGACCCCCACCTTGC	NM_214162	59	98
	GGAGGAACCACCGCCTGGGAT		60	
*MCP1 (CCL2; SCYA2*)	GGTCCTTGCCCAGCCAGATGC	NM_214214.1	60	89
	TCATCAGCCGCTGCATCGAGA		58	
*PAI-1 (SERPINE-1)*	CGGACCACGGTCAAGCAGGTG	NM_213910	60	147
	CACCAGAACCAGGCGCGTCA		60	
*IL-18*	ACCAGGGACATCAAGCCGTGT	NM_213997.1	58	118
	ACTGCCAGACCTCTAGTGAGGC		57	
*ACACB (ACC2)*	TGCCGTGTCCCTGTTTGGGC	NM_001206399.1	60	126
	AGGCGCACAGCACACTGCTC		60	
*FABP4*	GCAGATGACAGGAAAGTCAAGAGCA	NM_001002817.1	57	58
	CCTGTACCAGGGCGCCTCCA		60	
*HSL (LIPE)*	AGTGCCTATGCTGGCGGGGA	NM_214315.1	60	116
	CCCAGGCGGAGGTCTCGGAA		60	
*LPL*	GTCTCGGGCCCAGCAGCATT	NM_214286	59	150
	GCGTGGGCTCCAAGGCTGTA		59	
*CD36*	GCTGTGGCAGCTGTACCCCA	NM_001044622	59	135
	TGGATCCGGATAGCCCCACAAT		57	
*GLUT4 (SLC2A4)*	CGGCATGGGTTTCCAGTATG	NM_001128433.1	60	61
	CGCGAAGAGAAGGAAGACGTA		58	
*ATGL (PNPLA2)*	GGCGGAACGGCCTCCTGAAC	NM_001098605.1	60	121
	TTGGCTCCGGCCCTCTCCTC		60	

^*^Ta Annealing temperature°C.

### Statistical analysis

The experiment was a completely randomized design. Data were analysed using SAS (SAS Inst. Inc., Cary NC, U.S.A.). When the dependent variable under investigation was a trait of the sow, data were analysed using a fixed effects models in PROC GLM in SAS (SAS Inst. Inc., Cary NC, U.S.A) with effects for treatment, parity group and their two-way interaction included in the model. Sow was the experimental unit. Sow body weight and sow backfat at day of insemination were used as covariates when the dependent variable was sow weight, and sow backfat, respectively. A single degree of freedom contrast was used to compare treatment C with all other treatments. Differences between treatment means were compared using Duncan’s multiple range test. Results were considered statistically significant when P ≤ 0.05 and were considered as tendencies when 0.05 < P < 0.10.

Offspring daily live-weight gain, average daily feed intake, carcass traits and RNA data were analysed using a mixed model in PROC MIXED in SAS (SAS Inst. Inc., Cary NC, U.S.A) with treatment, gender, birth weight category (i.e. light, medium and heavy) and their interactions as fixed effects. Sow was included as a random effect. The number of pigs born alive was included as a covariate when significant. Data not normally distributed was log transformed.

## Results

### Trial design

Gestational length in pigs is approximately 115 days. Control (C) sows consumed a standard gestation diet (1.8 kg food/day (23.5 MJ DE/day) from day 1 to day 24 gestation, 2.3 kg food/day (30 MJ DE/day) from day 25 to 110 gestation, followed by 1.8 kg food/day (25.6 MJ DE/day) from day 111 to parturition (Table 2). This level of food intake provides sufficient energy for a 195 kg sow at day 1 gestation, to reach a target total gestational weight gain of approximately 50 kg and a backfat increase of 9 mm [30,31]. Overfeeding prior to day 25 gestation is not recommended due to an increased risk of embryo death [31,30]. As such, the earliest dietary intervention was at day 25 gestation. E sows consumed 4.6 kg/day food in early gestation (days 25 to 50 gestation) (Table 2). M sows received 4.6 kg/day in mid gestation (days 50 to 80) (Table 2). EM sows received 4.6 kg/day in early and mid gestation (days 25 to 80) (Table 2). L sows were fed to appetite (3.5 kg/day) from days 80 to 110 of gestation (Table 2). Diet composition did not differ across treatments.

### Maternal gestational weight gain trajectory

**Figure 1:**
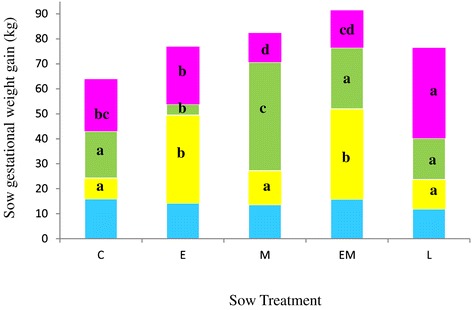
**Maternal weight gain during gestation.** Control C Sows (n = 10) received 2.3 kg/day food from day 25 to 110 gestation, E sows (n = 15) received 4.6 kg/day food in early gestation (days 25 to 50), M sows (n = 13) received 4.6 kg/day in mid gestation (days 50 to 80), EM sows (n = 12) received 4.6 kg/day in early and mid gestation (days 25 to 80) and L sows (n = 11) received 3.5 kg/day from days 80 to 110 of gestation. Colour coding; blue depicts weight gain from service to day 25 gestation, yellow depicts weight gain from day 25 to day 50 gestation, green depicts weight gain from day 50 to day 80 gestation and pink depicts weight gain from day 80 to day 110 gestation. Values represent Duncan’s means. Different superscripts indicate significant differences (P < 0.05) between treatments within the same time interval.

All sows had similar body weights at service (189.2 kg, SEM 1.1, P > 0.05). The effect of treatment on sow body weight during gestation did not differ by parity. At the end of gestation (day 110), only treatment EM sows were heavier than treatment C sows (275.8 kg versus 255.4 kg, SEM 5.09, P < 0.05). However, timing of gestational weight gain differed across treatments (Figure 1). From day 25 to 50 gestation, E and EM sows gained significantly more weight than treatment C, M and L sows (P < 0.05). From day 50 to 80 gestation, M sows gained significantly more weight than all other treatments (P < 0.05). In contrast, within this time period, E sows gained significantly less weight than all treatments (P < 0.05). From day 80 to 110 gestation, L sows gained significantly more weight than all other treatments (P < 0.05). Within this time period, C sows had similar weight gains to E and EM sows (P > 0.05) but significantly greater weight gains than M sows (P < 0.05). Treatment M had similar weight gains to EM sows (P > 0.05) (Figure 1).

### Maternal gestational backfat

All sows had similar backfat measurements at service (12.4 mm, SEM 0.29, P > 0.05) (Table 4). At day 50 of gestation, sows from treatment EM had greater backfat depth than C sows (P < 0.05). This was still evident at day 80 (P < 0.05) and day 110 gestation (P ≤ 0.05). Treatments M sows also had increased backfat depth than C sows at day 80 gestation (P < 0.05) and a tendency to have higher backfat at day 110 gestation (P < 0.1).

**Table 4:** **Effect of treatment on sow backfat during gestation**

	**Gestation feeding treatment** ^**a**^						**P-value**				
	**C**	**E**	**M**	**EM**	**L**	**SEM**	**Trt**	**C v E**	**C v M**	**C v EM**	**C v L**
No. of sows	10	15	13	12	11						
Sow backfat, mm											
At service	12.4	11.5	12.4	11.2	13.6	0.29	0.07	0.34	0.92	0.23	0.18
Day 25 gestation	13.1	12.4	13.4	12.7	14.2	0.74	0.52	0.57	0.78	0.73	0.34
Day 50 gestation	13.6	15.6	13.9	16.7	15.4	0.92	0.14	0.14	0.84	0.03	0.21
Day 80 gestation	14.1	16.3	17.5	17.9	15.3	1.02	0.09	0.14	0.03	0.02	0.45
Day 110 gestation	14.8	16.7	17.9	18.4	17.1	1.2	0.32	0.28	0.08	0.05	0.21

^a^C were Control Sows that received 2.3 kg/day food from day 25 to day 110 gestation, E sows consumed 4.6 kg/day food in early gestation (days 25 to 50), M sows received 4.6 kg/day in mid gestation (days 50 to 80), EM sows received 4.6 kg/day in early and mid gestation (days 25 to 80) and L sows received 3.5 kg/day from days 80 to 110 of gestation.

A P value of < 0.05 indicates significance.

### Offspring phenotypes

Offspring from this study were 159 days old at slaughter, corresponding to human adolescence [22]. Post weaning, offspring were given *ad libitum* access to food. The effect of treatment on offspring weight and body composition has been presented, in part, previously [27] and together with food intake data is summarized in Table 5. Birth weight of offspring was not affected by treatment of sows (P > 0.05). However offspring weight trajectories, food intake and body composition at slaughter were influenced by maternal food allocation during gestation (Table 5).

**Table 5:** **Effect of maternal treatment on offspring from birth to adolescence**

	Treatments*							**P VALUE**
	**C**	**E**	**M**	**EM**	**L**	**N** ^**†**^	**se**	
**WEIGHTS (kg)**								
Birth weight	1.53	1.45	1.47	1.47	1.53	47	0.033	0.25
Weaning weight^‡^	7.59c	8.22ab	8.34a	7.71bc	7.60c	47	0.19	<0.01
Weight Day 41	10.5	11.0	10.9	10.7	11.1	47	0.21	0.32
Weight Day 55	17.4	17.0	17.8	17.5	17.7	47	0.36	0.54
Weight Day 76	30.0	29.0	29.4	28.7	29.1	41	0.71	0.7
Weight Day 118	62.2a	56.1b	58.3ab	58.9ab	56.7b	34	1.48	<0.05
Weight Day 159	97.6a	90.0b	92.2ab	95.1ab	90.8b	34	1.87	<0.05
**DAILY FEED INTAKE (g)**								
Weaning to Day 41	286ab	277b	247c	280b	306a	47	8.0	<0.001
Day 41 to 55	787	726	740	730	757	47	24.9	0.41
Weaning to Day 55	550a	505ab	503b	514ab	539ab	47	15.2	0.10
Day 55 to 76	1158ab	1209a	1147ab	1094b	1119ab	45	31.3	0.13
Weaning to Day 76	821	819	775	766	794	41	20.1	0.13
Day 76 to 118	1781a	1608b	1674ab	1687ab	1693ab	34	41.6	0.08
Day 118 to 159	2449ab	2515a	2469ab	2439ab	2286b	34	61.6	0.10
Day 76 to 159	2120	2045	2063	2058	1986	34	43.8	0.42
Weaning to Day 159	1647	1599	1600	1583	1547	34	31.7	0.35
**SLAUGHTER** ^**§**^ **DATA**								
Carcass weight (kg)	73.6a	68.1b	68.7b	72.4ab	69.5ab	32	1.56	<0.05
Fat (mm)	10.9a	9.7bc	9.2c	11.0a	10.5ab	32	0.38	<0.01
Lean (%)	58.5bc	59.4ab	59.8a	58.3c	59.0abc	32	0.36	<0.01

*C = Control Sows consumed 2.3 kg/day food from day 25 to 110 gestation, E sows consumed 4.6 kg/day food in early (days 25 to 50) gestation, M sows consumed 4.6 kg/day in mid gestation (days 50 to 80), EM sows consumed 4.6 kg/day in early and mid gestation (days 25 to 80) and L sows received 3.5 kg/day from days 80 to 110 of gestation.

^†^N = number of Offspring.

^‡^Weaning of offspring occurred at day 28.

^§^Offspring were slaughtered at 159 days old.

Different superscripts, within the row, indicate significant differences.

P value < 0.1 is defined as a tendency and < 0.05 is significant.

There was a treatment by birth weight interaction for offspring food intake from day 41 to 55 and from weaning to day 76. There was a treatment by gender interaction for offspring food intake from all time points recorded from day 76 to 159 and overall from weaning to 159 days old. In addition, there was a treatment by gender interaction, at slaughter, for both offspring lean % and muscle depth with a tendency observed for backfat measurements.

At weaning, offspring born to E sows were heavier than offspring born to C sows (P < 0.05) (Table 5). From day 76 to 118 days old, treatment E offspring tended to have reduced food intake compared to treatment C offspring. As such on day 118 (P < 0.05) and on day 159 (P < 0.05), offspring born to E sows were lighter than offspring born to C sows. At slaughter, treatment E offspring had lighter carcass weight (P < 0.05) and reduced backfat (P < 0.01) than treatment C offspring.

Treatment M offspring were heavier than treatment C offspring at weaning age (P < 0.01). From weaning to day 41, treatment M offspring ate less than C offspring (P < 0.001). Although there was no difference in live weight at slaughter, M offspring had lighter carcass weight (P < 0.05) reduced backfat (P <0.01) and increased lean meat % (P < 0.01) compared to treatment C offspring.

Treatment EM offspring had a similar weight trajectory, food intake level and body composition to treatment C offspring.

Treatment L offspring were lighter than treatment C offspring on day 118 (P < 0.05) and on slaughter day (P < 0.05) but carcass weight, backfat thickness and % lean at slaughter were similar.

Offspring birth weight and gender effects have been discussed previously [27]. Briefly, offspring birth weight had a significant influence on offspring weight at all time points, food intake at all time points, except day 118 to 159, carcass weight and muscle depth. Male offspring tended to be heavier than female offspring at day 159 (94.5 kg versus 91.8 kg, SEM 1.42 P < 0.1). Gender influenced food intake with males having a reduced food intake compared to females from all time points from day 55 to 159 and overall from weaning to day 159 (1570 g versus 1624 g, SEM 21 P < 0.01). Males had greater backfat depth, less muscle depth and reduced % lean than females [27].

### Offspring subcutaneous adipose signals

**Figure 2:**
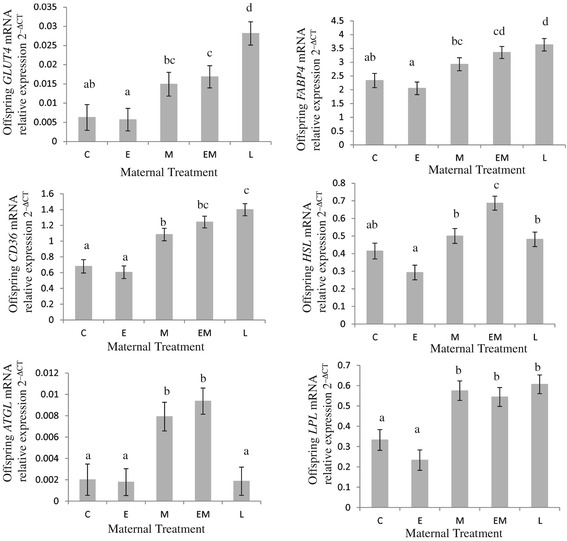
**Messenger RNA levels of** ***GLUT4*** **,** ***FABP4*** **,** ***CD36*** **,** ***HSL*** **,** ***ATGL*** **and** ***LPL*** **in offspring subcutaneous adipose tissue.** Maternal treatments: Control C Sows received 2.3 kg/day food from day 25 to 110 gestation, E sows received 4.6 kg/day food in early (days 25 to 50) gestation, M sows received 4.6 kg/day in mid gestation (days 50 to 80), EM sows received 4.6 kg/day in early and mid gestation (days 25 to 80) and L sows received 3.5 kg/day from days 80 to 110 of gestation. Data was generated from n = 18 adipose samples per treatment. At least 2 experimental repeats and 4 technical repeats were performed on each adipose sample. Relative amount of mRNA target = 2^–ΔCT^ where ΔCT = crossing threshold of Target - crossing threshold of reference *rPLPO*. Different superscripts within a figure indicate significant differences between treatments, P < 0.05.

**Figure 3:**
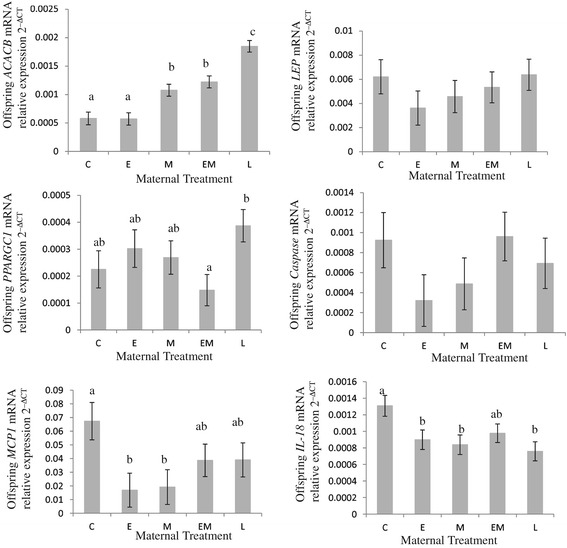
**Messenger RNA levels of** ***ACACB*** **,** ***LEP*** **,** ***PPARGC1*** **,** ***Caspase*** **,** ***MCP1*** **and** ***IL-18*** **in offspring subcutaneous adipose tissue.** Maternal treatments: Control C Sows received 2.3 kg/day food from day 25 to 110 gestation, E sows received 4.6 kg/day food in early (days 25 to 50) gestation, M sows received 4.6 kg/day in mid gestation (days 50 to 80), EM sows received 4.6 kg/day in early and mid gestation (days 25 to 80) and L sows received 3.5 kg/day from days 80 to 110 of gestation. Data was generated from n = 18 adipose samples per treatment. At least 2 experimental repeats and 4 technical repeats were performed on each adipose sample. Relative amount of mRNA target = 2^–ΔCT^ where ΔCT = crossing threshold of Target - crossing threshold of reference *rPLPO*. Different superscripts within a figure indicate significant differences between treatments, P < 0.05.

**Figure 4:**
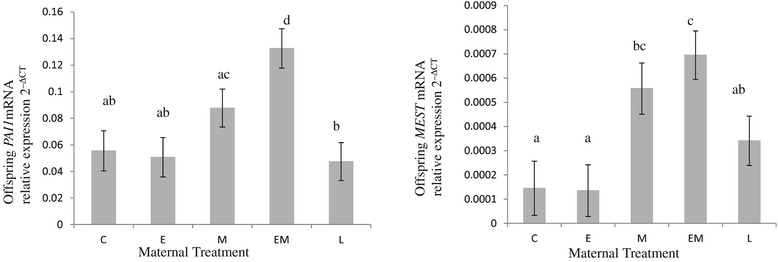
**Messenger RNA levels of** ***PAI1*** **and** ***MEST*** **in offspring subcutaneous adipose tissue.** Maternal treatments: Control C Sows received 2.3 kg/day food from day 25 to 110 gestation, E sows received 4.6 kg/day food in early (days 25 to 50) gestation, M sows received 4.6 kg/day in mid gestation (days 50 to 80), EM sows received 4.6 kg/day in early and mid gestation (days 25 to 80) and L sows received 3.5 kg/day from days 80 to 110 of gestation. Data was generated from n = 18 adipose samples per treatment. At least 2 experimental repeats and 4 technical repeats were performed on each adipose sample. Relative amount of mRNA target = 2^–ΔCT^ where ΔCT = crossing threshold of Target - crossing threshold of reference *rPLPO*. Different superscripts within a figure indicate significant differences between treatments, P < 0.05.

To determine whether adipose signals were different in offspring from mothers with different gestational weight gain patterns, mRNA levels of a panel of genes were quantified in subcutaneous adipose tissue harvested from adolescent offspring. Figures 2, 3 and 4 detail levels of lipid metabolism, adipocyte hypertrophy and inflammation mRNA markers, compared to ribosomal *rPLPO* mRNA levels, in offspring subcutaneous adipose tissue. Levels of mRNA *GLUT4*, which codes for the major glucose transporter in adipocytes [36] were higher in treatment EM (2.8 fold increase, P < 0.05) and L offspring (4.6 fold increase, P < 0.001) compared to treatment C offspring (Figure 2). Levels of mRNA *FABP4*, which codes for a major lipid transport protein in mature adipocytes [37] were elevated in offspring born to treatment EM (1.5 fold increase, P < 0.01) and L (1.5 fold increase, P < 0.01) mothers compared to their control counterparts (Figure 2). *CD36* codes for a membrane glycoprotein which binds long chain fatty acids for transport into cells [38]. *CD36* mRNA levels were increased in treatment M (1.6 fold increase P < 0.01), EM (1.8 fold increase, P < 0.0001) and L (2 fold increase, P < 0.0001) offspring compared to treatment C offspring (Figure 2). *HSL* and *ATGL* code for enzymes that regulate the hydrolysis and mobilization of triacylglycerol stored in adipose cells into non-esterified fatty acids and glycerol for circulation [39]. *HSL* mRNA levels in treatment EM offspring were higher compared to offspring born to control sows (1.7 fold increase, P < 0.001) (Figure 2). *ATGL* mRNA levels were elevated in treatment M (4 fold increase, P < 0.01) and EM (4.5 fold increase, P < 0.01) offspring compared to treatment C offspring (Figure 2).

The lipogenic markers, *LPL* and *ACACB* [40,41], were also different in offspring adipose from different maternal treatments (Figures 2 and 3). *LPL* mRNA levels in subcutaneous adipose from offspring born to treatment M (1.75 fold increase, P < 0.001), EM (1.6 fold increase, P < 0.01) and L (1.8 fold increase, P < 0.001) mothers were higher than those in offspring born to control sows (Figure 2). Levels of *ACACB* mRNA were also elevated in treatment M (1.8 fold increase, P < 0.01) and EM (2.1 fold increases, P < 0.0001) offspring compared to controls, with treatment L offspring having the highest levels of *ACACB* mRNA in subcutaneous adipose samples (3.2 fold increase from levels observed in treatment C offspring, P < 0.0001) (Figure 3).

*Leptin (LEP)* and *CASPASE1* mRNA levels in offspring subcutaneous adipose tissue were unaffected by treatment of mother (Figure 3). *CASPASE1* codes for an enzyme involved in the activation of the inflammasome resulting in an increased flux of macrophages into adipose tissue [42]. *PPARGC1A* codes for a versatile transcriptional coregulator, that links nutritional signals to energy metabolism [43]. The subcutaneous adipose tissue of EM offspring had significantly less *PPARGC1A* mRNA transcripts than subcutaneous adipose tissue from L offspring (P < 0.01) (Figure 3).

Offspring born to treatment E (4 fold decrease, P < 0.01) and M (3,5 fold decrease, P < 0.05) had lower levels, in adipose tissue, of the gene coding for Monocyte Chemoattractant Protein-1 (*MCP1*) than offspring born to treatment C (Figure 3). *MCP1* codes for a cytokine with adipogenic functions and mRNA levels are positively correlated with obesity and insulin de-regulation [44].

The cytokine IL-18 is produced by non fat cells in adipose tissue [45]. There was a gender by treatment interaction for *IL-18* mRNA levels in subcutaneous adipose tissue. Treatment E (1.5 fold decrease, P < 0.05), treatment M (1.6 fold decrease, P < 0.05) and treatment L (1.7 fold decrease, P < 0.01) offspring had lower levels of *IL-18* mRNA in their subcutaneous adipose tissue than offspring from treatment C (Figure 3). Treatment EM offspring had elevated levels of *PAI-1* mRNA (2.4 fold increase, P < 0.001) in their subcutaneous adipose tissue compared to treatment C offspring (Figure 4). This inflammatory adipokine and angiogenic factor is primarily released by non-fat cells in adipose tissue [46]. The adipocyte size marker and imprinted gene [47], *MEST*, was increased in treatment M (3.8 fold increases, P < 0.05) and EM (4.8 fold increase, P < 0.01) offspring compared to treatment C offspring (Figure 4).

Gender influenced *CD36* mRNA levels in subcutaneous adipose tissue with females exhibiting 1.3 fold higher levels than males (P < 0.001) (data not shown). Subcutaneous adipose tissue of females also had higher levels of *FABP4* (1.3 fold increase, P < 0.001) and *GLUT4* (1.7 fold increase, P < 0.01) mRNA than males (data not shown).

Birth weight influenced levels of *IL-18* mRNA in subcutaneous adipose tissue with heavy and medium birth weight individuals exhibiting lower levels than low birth weight offspring (1.3 and 1.25 fold decrease, P < 0.05 respectively) (data not shown).

## Discussion

Increasing maternal food allowance in late gestation shifted the offspring subcutaneous adipocyte focus to accumulation of fat. Doubling maternal food allowance for 55 days in gestation, combining both early and mid gestation time periods, elevated lipogenic, nutrient transporters, lipolysis and adipocyte size markers in offspring subcutaneous adipose tissue. This adds credence to the suggestion that strict adherence to gestational weight gain guidelines are required for healthy weight mothers to protect their offspring against adipocyte dysfunction [2,5].

Doubling food allowances at different times of gestation did not result in ‘fat’ mothers per se, as backfat increases remained within the normal range (<9 mm backfat increase with a measurement of < 21 mm at farrowing) [31]. Although control sows gained 63.9 kg by day 110 of 115 days gestation, the backfat gain was less than expected at a modest 2.4 mm. However, and as expected, mothers from different treatments differed in their gestational weight gain patterns. In agreement with other studies, overfed mothers did not result in offspring birth weight differences [48-50].

### Effect of overfeeding mothers in late gestation

The largest maternal weight gain in late gestation occurred in treatment L mothers (36.2 kg from day 80 to 110 gestation). Excess nutrition during late gestation is predicted to exert the greatest influence on subcutaneous adipose function as the timing coincides with its rapid development, differentiation and maturation. Offspring born to treatment L sows had similar weight at birth to controls but were lighter by adolescence (day 118 and 159 day). However carcass weight, backfat thickness and % lean were unchanged for treatment L offspring suggesting lighter weights of internal organs and/or visceral fat. The subcutaneous adipose depot from treatment L offspring exhibited a bias towards fat storage and nutrient transport. Increased food intake in late gestation increased mRNA levels of all the lipogenesis markers tested (*CD36, ACACB* and *LPL*) with *ACACB* mRNA levels significantly higher in treatment L offspring than all other offspring in the study. Significantly higher *GLUT4* mRNA levels in the subcutaneous adipose tissue of treatment L offspring compared to all other adipose samples suggests increased levels of glucose entering the L adipocyte. Elevated levels of *FABP4* mRNA suggests more non-esterified fats may be transported to adipocyte membranes [37]. Although mRNA of the major lipolytic enzyme, HSL, was elevated in treatment L offspring subcutaneous adipose compared to control offspring, mRNA of the rate limiting enzyme ATGL which governs triacylglycerol store mobilization was not. No differences were observed for adiposity, adipocyte size and inflammation indicators. Over and undernutrition in late gestation in sheep interfered with offspring subcutaneous to visceral fat ratios [14]. Pregnant ewes fed 150% food allocation from day 115 gestation to term (day 150) gave birth to offspring who had similar body weights and growth rates from birth to day 30 but increased subcutaneous fat mass (40 g/kg versus 22.1 g/kg) [51]. However, indicators of lipogenesis (*PPARy, LPL* and *G3PDH* mRNA) remained unchanged [51]. Although discrepancies are evident with our study, nutritional intervention in late gestation in both studies did indicate enhanced fat storage ability in the subcutaneous adipose depot.

Offspring from our study were fed *ad libitum*. However, it can be postulated that a high fat or energy dense dietary challenge in adult offspring would amplify the fat storage bias. Rats born to overfed mothers had increased body weights, adipose weights, serum glucose, serum insulin, serum leptin, serum non esterified fatty acids and adipose triglycerides compared to controls. The introduction of a cafeteria diet from weaning to adulthood significantly amplified these differences [10].

### Effect of overfeeding mothers in both mid and early gestation

Doubling food intake in both early and mid gestation time periods, resulted in the heaviest and fattest mothers at parturition. EM mothers gained 36.2 kg in early gestation (day 25–50). This was followed in mid gestation by similar weight gains to control mothers leading to an overall gestational weight gain of 91.4 kg with a total backfat increase of 7.2 mm. Such backfat gains equate to a similar study where mothers received 42 MJ DE/ day from day 21 gestation to parturition [48]. In both studies, offspring born to overfed mothers with significant gestational weight gains did not differ to control offspring in birth weight, weight gain from birth to adolescence, food intake and carcass traits at adolescence [48]. Only when challenged with a high energy diet post-weaning, did Arentson-Lantz *et al.* observe increased fasting glucose and insulin plasma levels in 84 day old offspring whose mothers were overfed compared to controls [48]. At the transcriptional level, our EM offspring subcutaneous adipose tissue differed significantly from controls with increased levels of all lipogenic (*CD36*, *ACACB* and *LPL*), nutrient transporters (*FABP4* and *GLUT4*) and lipolysis (*HSL* and *ATGL*) indicators tested. Noticeably, *HSL* levels were the highest reported in the study. Adipose dysfunction is also indicated by elevated levels of *MEST* and *PAI-1* compared to controls [18]. However, this adipocyte dysfunction may require a high fat or energy dense dietary challenge to affect body weight parameters.

The longer length of the dietary intervention as well as the timing may have contributed to the significant differences observed. Long *et al.* reported that foetal subcutaneous adipose tissue from multiparous ewes, who received 150% food allocation above requirements throughout gestation, had elevated levels of *CD36, GLUT4, FASN, ACC* and *CD36* mRNA with accompanying increases in proteins CD36, FATP1, FATP4, GLUT4 and altered fatty acid composition compared to controls, although body weight was unchanged [52]. In agreement with our study, no differences in body weight from birth to adulthood were observed in female offspring born to ewes who received 200% food allowance in early and 140% food allowance in mid gestation compared to those who received the recommended allowance [53]. In addition, Munoz *et al.* 2009 observed that the male offspring born to overfed ewes had similar carcass weight, fat depth, retroperitoneal fat level and perinephric fat level to control offspring [53]. Gestational overnutrition via maternal intragastric cannulation resulted in rats, at weaning age, of similar body weight and % fat mass but enhanced hepatic lipogenic signalling compared to control offspring [20]. Adult offspring born to sows on a high protein gestational diet (30%) but normal food intake levels (34 MJ DE/day) exhibited no differences in body weight, backfat thickness, perirenal fat %, ommental fat %, adipocyte area and *LEP* mRNA levels in subcutaneous adipose tissue compared to controls [54]. However metabolic and lipid transport differences were observed in the proteome of the backfat subcutaneous adipose depot of these offspring as early as 1 day old, compared to their controls [55].

In humans there is a well-recognised association between maternal gestational weight gain and infant birth weight [56-58]. In addition Hull *et al.* reported that mothers with a healthy BMI pre-pregnancy, who gained more than the recommended weight during gestation, gave birth to infants with similar infant fat mass but greater fat free mass to mothers with an appropriate gestational weight gain [59]. In other studies, mothers who gained more than 16 kg during pregnancy increased the odds ratio of their children being overweight by the age of 8 years, although the mothers BMI pre-pregnancy was not accounted for [60]. Wrotniak *et al.* reported a significant increase in the number of children overweight at the age of 7 when their healthy BMI mothers gained excessive weight during pregnancy compared to mothers of similar BMI who gained appropriate weight [58]. Excessive gestational weight gain in healthy pre-pregnancy weight mothers increased the odds ratio by 1.5 fold for greater adiposity in adult daughters [57].

### Effect of overfeeding mothers in mid gestation

Doubling food allowance in mid gestation resulted in a 43.5 kg weight gain between days 50 and 80 compared to 18.7 kg gain in control sows for the same time period. However, these M sows had a reduced weight gain in late gestation compared to controls (11.69 kg versus 20.9 kg). Treatment M offspring demonstrated early postnatal weight gain during the suckling period. At slaughter, these M adolescent offspring had lighter carcass weight, reduced backfat and increased lean meat % compared to treatment C offspring. Subcutaneous adipose tissue in treatment M offspring exhibited a fat storage rather than a lipogensis bias. Increased maternal food intake during foetal subcutaneous lipid deposition may alter fat cell ability to store and release fatty acids. Fat storage indicators (*CD36, LPL* and *ACACB*) and the adipocyte size marker *MEST* were upregulated in treatment M offspring compared to their control counter parts. Lower levels of *MCP1* and *IL-18* implied a lower level of T-lymphocyte and macrophage infiltration [44,45]. This agreed with lower backfat measurements as there is positive association between these markers and adiposity levels [44,45]. However, there is a disconnect between the lipogenic bias of the subcutaneous adipose depot and the unchanged *LEP* mRNA levels and reduced backfat level. Although measurement of mRNA levels of lipogenic genes is a reasonable indication of lipogenic activity [61], the actual lipogenic activity of the subcutaneous adipocyte was not measured. In a previous study, increased levels of *CD36* and *GLUT4* mRNA paralleled with increased levels CD36 and GLUT4 peptide in subcutaneous adipose tissue of lamb foetuses whose mothers were overfed 150% requirements from 60 days pre conception to term [52]. Male lamb foetuses in late gestation (day 135 of 150 days gestation) had lighter carcass weights (3.56 kg versus 4.12 kg) [52]. In agreement with our study, providing cows with extra nutrition in mid gestation (day 135–195 gestation) resulted in male offspring with similar birth weights but increased weaning weights (256 kg versus 242.1 kg) compared to controls [62]. By slaughter age, these offspring had increased average daily gain (1.656 kg/day versus 1.489 kg/day), live weight (543.9 kg versus 520.6 kg), 12th rib fat thickness (1.51 cm versus 1.11 cm) and hot carcass weight (348 kg versus 330 kg) compared to control offspring [62]. There was no difference in longissimus muscle area or fibre type but there was a tendency to have increased number of subcutaneous adipocytes [62]. In our study, increased food allowance in mid gestation did not affect offspring total fibre number, type, or secondary to primary fibre ratio [26,27]. Therefore, although treatment M delivers increased maternal nutrition at a time when offspring secondary muscle fibres are formed, the increased lean observations is probably a direct result of offspring fat depth. In contrast, Ceriuselo *et al.* reported a reduction in porcine muscle fibre number, particularly type IIB, in adult offspring of mothers overfed (60 MJ ME/day) from day 45 to 85 gestation [50]. In agreement with our study, overfeeding in mid gestation did transiently alter food intake and average daily gain measurements during growth but adolescent offspring had similar body weight, carcass weight and lean % to controls [50].

The significantly lower weight gain in the last trimester at a time of rapid adipose expansion may have also contributed to the offspring reduced backfat and elevated lipogenic biomarkers. Undernutrition in the last trimester resulted in offspring with reduced subcutaneous backfat layer in lamb offspring at adolescence (6 months of age) [63] and reduced subcutaneous fat pads in young rat offspring (24 days old) [61].

### Effect of overfeeding mothers in early gestation

Doubling food in early gestation resulted in a 35 kg weight gain from day 25 to 50 gestation compared to 8 kg in control sows for the same time period. However this early and substantial weight gain was followed by a marginal weight gain in mid gestation. Offspring born to these E sows had different postnatal growth rates compared to C offspring. At adolescence, E offspring were 7.6 kg lighter with 1.2 mm less backfat that offspring born to C sows. However lipogenesis and lipolysis markers in subcutaneous adipose tissue of E offspring remained similar to control individuals. In addition, no differences in *MEST* mRNA levels or *LEP* mRNA would suggest no difference in adiposity or adipocyte size [47]. This indicates that subcutaneous adipocyte function is unchanged to controls. Early gestational weight gain is unlikely to impact on the metabolism of offspring subcutaneous adipose tissue as porcine subcutaneous fat cell differentiation begins after day 45 gestation [28]. Instead extra nutrition in early gestation may alter (a) skeletal muscle physiology and/or (b) number of committed adipocyte precursor cells to either subcutaneous or visceral fat depots. Although treatment E offspring had similar % lean and muscle depth to C offspring, we previously observed that E offspring had increased semitendinosis type II oxidative muscle fibres [26], *calcenurin* mRNA [27] and reduced semimembranous intramuscular fat [27]. Previous studies of over nutrition, under nutrition or low protein diet in early gestation resulted in modifications to muscle weight [64], fibre number [49], adipose yield [49], subcutaneous and visceral fat levels [12,64-66].

## Conclusions

In conclusion, increasing gestational weight gain in early gestation altered offspring postnatal growth rate. Increasing maternal food allowance in late gestation appeared to shift the offspring adipocyte focus towards fat accretion. Increasing maternal food allowance in early and mid gestation combined, resulted in offspring whose subcutaneous adipose tissue, at adolescence, exhibited elevated mRNA levels of lipogenic, nutrient transporters, lipolysis and adipocyte size indicators compared to controls. The present study provides additional evidence that mothers, who gain more than the recommended weight gain in mid and late gestation, put their offspring adipose tissue at risk of dysfunction.

## Abbreviation

BMI: Body mass index
